# Decline in the incidence of invasive pneumococcal disease at a medical center in Taiwan, 2000–2012

**DOI:** 10.1186/1471-2334-14-76

**Published:** 2014-02-11

**Authors:** Chih-Cheng Lai, Sheng-Hsiang Lin, Chun-Hsing Liao, Wang-Huei Sheng, Po-Ren Hsueh

**Affiliations:** 1 Department of Intensive Care Medicine, Chi Mei Medical Center, Liouying, Tainan, Taiwan; 2 Department of Respiratory Therapy, Fu Jen Catholic University, New Taipei City, Taiwan; 3 Department of Internal Medicine, New Taipei City Hospital, New Taipei City, Taiwan; 4 Department of Internal Medicine, Far Eastern Memorial Hospital, New Taipei City, Taipei County, Taiwan; 5 Department of Internal Medicine, National Taiwan University Hospital, National Taiwan University College of Medicine, Taipei, Taiwan; 6 Department of Laboratory Medicine, National Taiwan University Hospital, National Taiwan University College of Medicine, Taipei, Taiwan

**Keywords:** Invasive pneumococcal disease, Incidence, *Streptococcus pneumoniae*, Serotypes

## Abstract

**Background:**

It is essential to investigate the serotype distribution of pneumococcal diseases in each region and its associated clinical features. This study investigated the annual incidence of invasive pneumococcal disease (IPD) and the distribution of serotypes of isolates causing IPD at a medical center in northern Taiwan during the period 2000 to 2012.

**Methods:**

Serotypes of all available *Streptococcus pneumoniae* isolates causing IPD were determined using the latex agglutination test.

**Results:**

During the study period, the annual incidence (per 10,000 admissions) of IPD decreased significantly from 9.8 in 2000 to 2.1 in 2012 (P < 0.001). The annual incidence of all-cause bacteremia, primary pneumococcal bacteremia, bacteremic pneumonia, peritonitis, and meningitis also decreased significantly during the study period (*P* < 0.05). In contrast to the decrease in annual incidence of pneumococcal serotypes 14, 23F and 6B, the incidence and the proportion of serotype 19A significantly increased with time (*P* < 0.001). The coverage rate of 7-valent protein conjugated vaccine (PCV-7) and PCV-10 decreased significantly; however, the coverage rate of PCV-13 and pneumococcal polysaccharide vaccine (PPV-23) remained stable over time. Serotype 14 and 19A isolates were commonly isolated from blood and pleural effusion, respectively. Serotypes 14 and 23F were the two most common serotypes found in adult patients, and serotypes 14 and 19A were the two most common serotypes isolated from children.

**Conclusions:**

Although the incidence of IPD has decreased, serotype 19A is an emerging problem in Taiwan. The distribution of serotypes of pneumococci varied with clinical symptoms and age. As the changing distribution of pneumococcal serotype with time, the coverage rate of pneumococcal vaccines would be different.

## Background

*Streptococcus pneumoniae* is a well known etiology of acute otitis media, pneumonia, bloodstream infections, peritonitis, and meningitis [1-5]. Although there are 92 different capsular serotypes, which have been categorized into 46 serogroups based on immunological characteristics [6], less than 30 types account for more than 90% of invasive pneumococcal disease (IPD) [7]. Different serotypes have distinct features, which may be more frequently associated with specific disease patterns. For example, serotypes 3, 6B, 14, 23F can cause more severe meningeal inflammation than serotypes 1, 5, 9, and 7F in experimental meningitis [8,9]. In a murine model of pneumococcal sepsis, certain serotypes were more fatal than other serotypes [10]. Moreover, the distribution of serotypes varies according to patient’s age, disease pattern, and geographical region [1].

Although the mortality rate associated with IPD is as high as 35% [11-14], pneumococcal vaccination was developed to prevent several serotypes. Furthermore, serotype distribution can be affected by the implementation of vaccination [1]. Most important of all, novel vaccine development should be based on knowledge of the incidence rate of each pneumococcal serotype. When facing this life-threatening disease, it is essential to investigate the serotype distribution in each region and its associated clinical features. Therefore, we investigated the serotype distribution of various types of IPD and the trend with time from 2000 to 2012 at a medical center in Taiwan.

## Methods

### Hospital setting and patient selection

This study was conducted at the National Taiwan University Hospital (NTUH). NTUH has a 2500-bed and around 8000 daily clinical visits, and is a both primary care and tertiary care center in northern Taiwan. Patients whose blood, cerebrospinal fluid (CSF), pleural effusion, ascites, or aspirated ear and sinus fluid cultures yielded *S. pneumoniae* during 2000–2012 were identified from the computerized database of the bacteriology laboratory.

### Bacterial isolates and serotype study

Pneumococcal isolates were identified by recognition of typical colony morphology on trypticase soy agar supplemented with 5% sheep blood (BBL Microbiology Systems, Cockeysville, MD), Gram staining characteristics, susceptibility to ethylhydrocupreine hydrochloride (optochin) (Difco Laboratories, Detroit, MI) and bile solubility. Serotypes were determined for available isolates by latex agglutination (Pneumotest-Latex; Statens Serum Institut, Copenhagen, Denmark) according to the manufacturer’s instructions and were confirmed by the Quellung reaction [15,16].

### Definition

IPD was diagnosed in patients from whom *S. pneumoniae* was isolated from a normally sterile site, such as blood, cerebrospinal fluid, pleural fluid, or ascites. Pneumococcal meningitis was diagnosed in patients from whom pneumococci were isolated from CSF. The diagnoses of pneumococcal empyema, peritonitis, sinusitis, and otitis media were established based on culture results positive for *S. pneumoniae* obtained from pleural fluid, ascites, aspirated sinus, and ear fluid, respectively. Primary pneumococcal bacteremia was defined as isolation of pneumococcus from blood without a focus. The annual incidence of each type of IPD was defined as the number of patients with pneumococcal infections per 10,000 admissions per year.

### Statistical analysis

The chi-square test was used for dichotomous variables. We used the chi-square test for trend to assess temporal trends in incidence. A P value of < 0.05 was considered to be statistically significant.

## Results

### Secular trend of incidence of IPD

**Figure 1:**
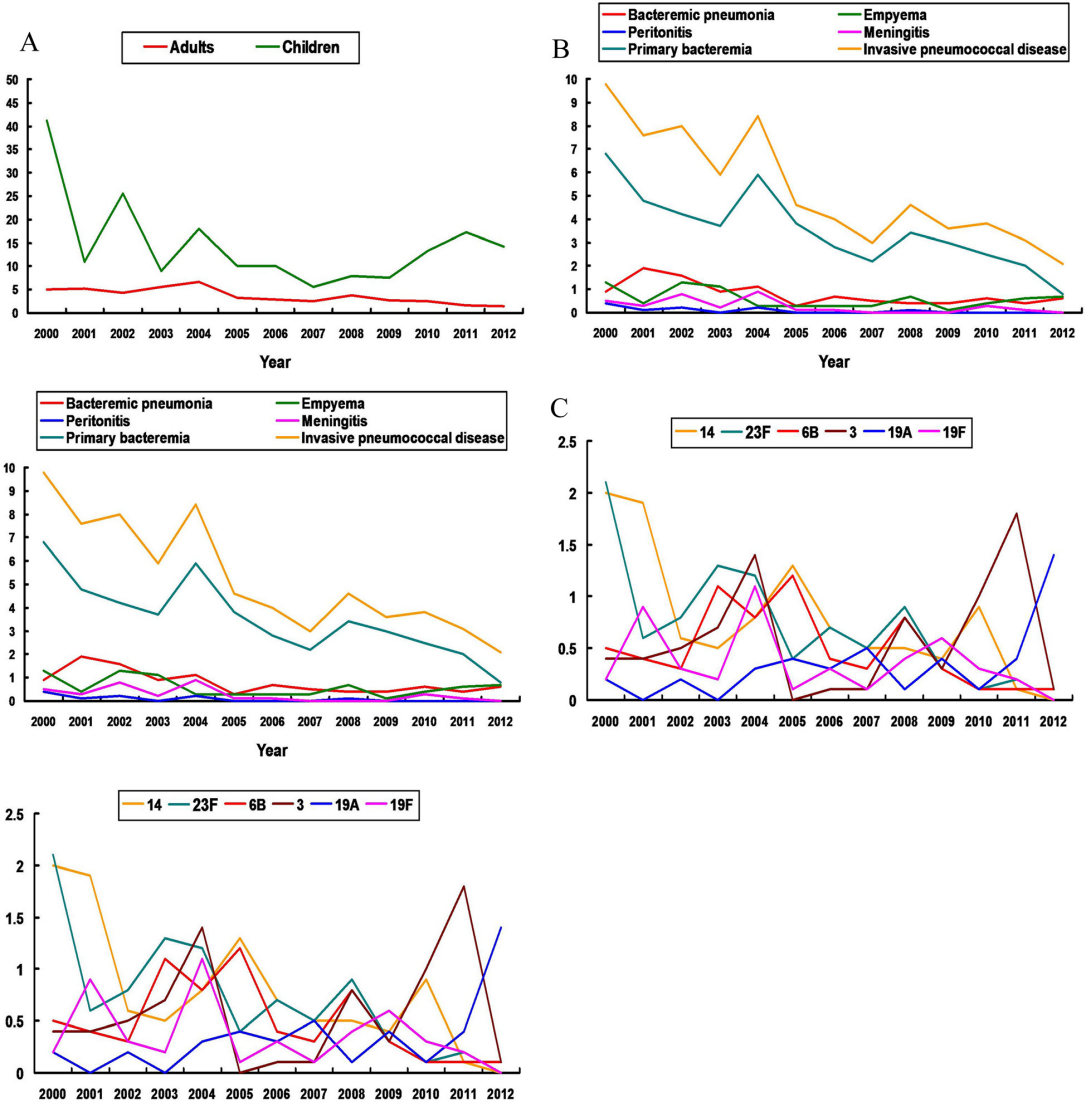
**Annual incidence (number of patients per 10,000 admissions) of invasive pneumococcal disease (IPD). (A)** Annual incidence of IPD among adults and children, major type of IPD **(B)**, and major pneumococcal serotype **(C)**.

During the study period, IPD was diagnosed in 458 patients and of those patients, 423 had bacteremia. Empyema was found in 52 (11.4%) patients, peritonitis was found in 22 (4.8%) patients, and meningitis was found in 6 (1.3%) patients. Additionally, 25 cases of culture-confirmed sinusitis and 25 cases of culture-confirmed otitis media were identified during the study period. The yearly admission number gradually increased with time (*P* < 0.05), ranging from 55,920 in 2000 to 85,253 in 2012 (Table 1). The annual incidence of each type of IPD per 10,000 admissions is shown in Table 1 and Figure 1A and B. In children, the annual incidence of IPD significantly decreased from 41.2 per 10,000 admissions in 2000 to 15.2 per 10,000 admissions in 2012 (*P* < 0.001). In adult, the annual incidence of IPD significantly decreased from 5.0 per 10,000 admissions in 2000 to 1.5 per 10,000 admissions in 2012 (*P* < 0.001). The overall annual incidence of IPD significantly decreased from 9.8 per 10,000 admissions in 2000 to 2.1 per 10,000 admissions in 2012 (*P* < 0.001). Additionally, the annual incidence of all-cause bacteremia, primary bacteremia, bacteremic pneumonia, peritonitis, and meningitis due to *S. pneumoniae* significantly decreased with time (*P* < 0.05). The annual incidence of empyema, pneumonia without bacteremia, sinusitis and otitis media remained stable between 2000 and 2012. Figure 1C shows the annual incidence of major serotypes of *S. pneumoniae* causing IPD. The annual incidence of serotypes 14, 23F and 6B significantly decreased with time; however, the incidence of serotypes 19A significantly increased (P <0.001). There was no significant change in annual incidence of serotypes 3 and 19F (*P* = 0.172 and *P* = 0.08).

**Table 1:** Annual incidence of patients with each type of invasive pneumococcal disease (IPD), sinusitis, and otitis media at National Taiwan University Hospital from 2000 to 2012

**Disease**	**Incidence expressed as no. of patients per 10,000 admission (no. of patients)**													***P *** **value**
	**2000 (n = 55920)**	**2001 (n = 66743)**	**2002 (n = 62177)**	**2003 (n = 54574)**	**2004 (n = 64186)**	**2005 (n = 67853)**	**2006 (n = 70814)**	**2007 (n = 73534)**	**2008 (n = 74505)**	**2009 (n = 78756)**	**2010 (n = 79710)**	**2011 (n = 81594)**	**2012 (n = 85253)**	
IPD	9.8	7.6	8.0	5.9	8.4	4.6	4.0	3.0	4.6	3.6	3.8	3.1	2.1	<0.001
All bacteremia	9.3	7.3	7.1	5.1	7.9	4.1	3.8	2.9	4.2	3.4	3.4	2.7	1.9	<0.001
Primary bacteremia	6.8	4.8	4.2	3.7	5.9	3.8	2.8	2.2	3.4	3.0	2.5	2.0	0.8	<0.001
Bacteremic pneumonia	0.9	1.9	1.6	0.9	1.1	0.3	0.7	0.5	0.4	0.4	0.6	0.4	0.6	<0.001
Empyema	1.3	0.4	1.3	1.1	0.3	0.3	0.3	0.3	0.7	0.1	0.4	0.6	0.7	0.070
Peritonitis	0.4	0.1	0.2	0.0	0.2	0.0	0.0	0.0	0.1	0.0	0.0	0.0	0.0	< 0.009
Meningitis	0.5	0.3	0.8	0.2	0.9	0.1	0.1	0.0	0.0	0.0	0.3	0.1	0.0	<0.001
Non-IPD														
Pneumonia without bacteremia	16.8	18.4	24.1	19.1	23.2	23.4	27.1	23.0	25.6	22.1	23.5	15.0	12.7	<0.054
Sinusitis	0.2	0.3	0.3	0.2	0.6	0.3	0.3	0.4	0.4	0.0	0.3	0.1	0.2	0.439
Otitis media	0.4	0.3	0.2	0.2	0.5	0.3	0.3	0.4	0.3	0.3	0.0	0.2	0.4	0.69

### Serotype distribution in adults and children

**Figure 2:**
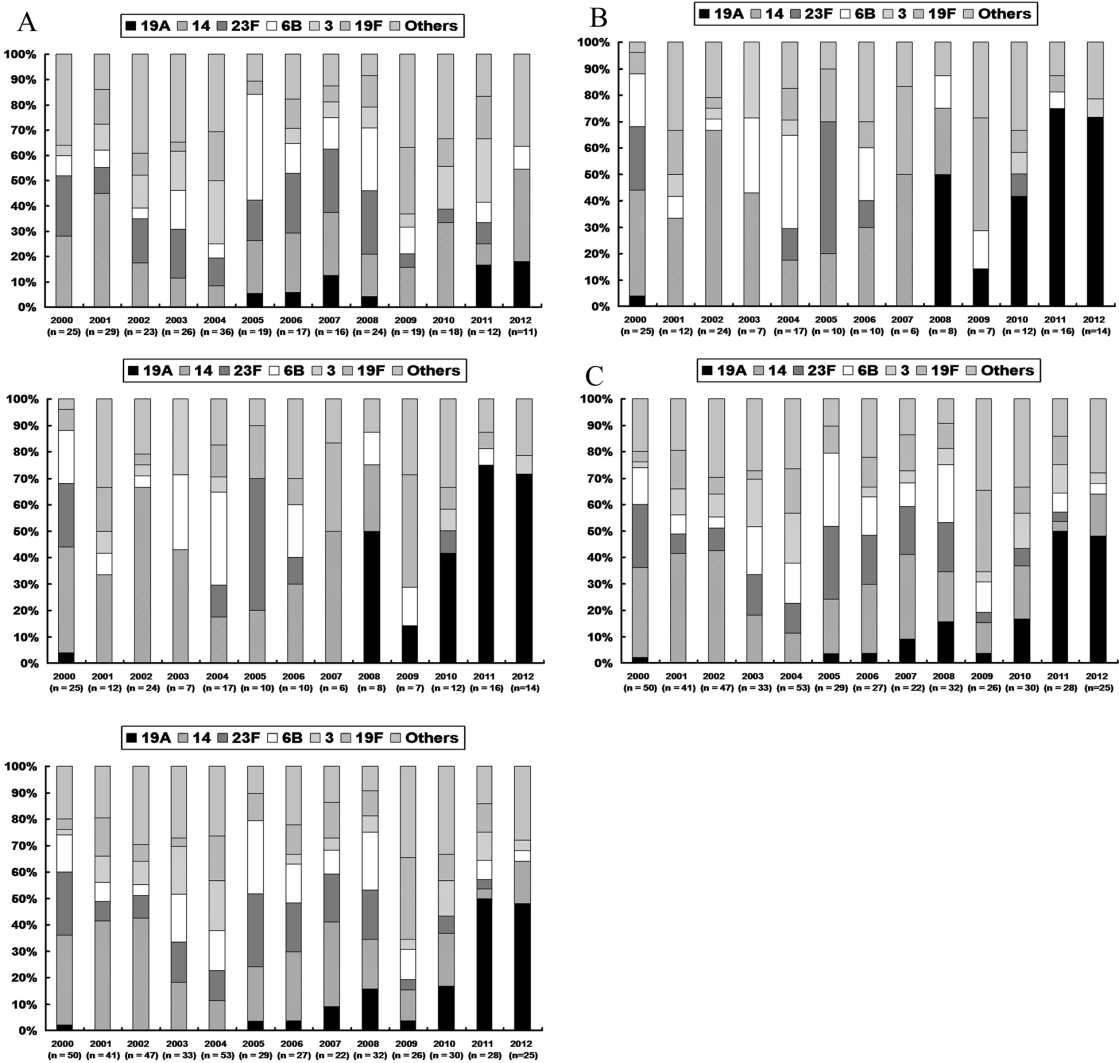
**Distribution of serotypes among *S. pneumoniae *isolates. (A)**, children **(B)** and all patients with invasive pneumococcal diseases **(C)**.

During the study period, a total of 443 isolates from 443 patients with IPD were available for serotype studies. The annual distribution of main capsular serotypes of *S. pneumoniae* isolates causing IPD is shown in Figure 2. For all patients, the proportion of serotype 19A increased from <20% between 2000 and 2007 to 48.0%–50.0% between 2011 and 2012 (*P* < 0.001). Table 2 shows the serotype distribution in adults, children, and all patients. Among all patients, serotype 14 was the most common serotype (n = 106, 23.9%), followed by serotype 23F (n = 57, 12.9%), and serotype 6B (n = 53, 12.0%). Serotype 14 was also the most common serotype found in adult patients, followed by serotype 23F, and 6B. In contrast, the second most common serotype in children was serotype 19A, followed by serotype 19F.

**Table 2:** **Distribution of major serotypes of 443 isolates of *S. pneumoniae *causing invasive pneumococcal disease in adults and children treated National Taiwan University Hospital from 2000 to 2012**

**Serotype**	**Adults (n = 275)**		**Children (n = 168)**		**Total (n = 443)**	
	**No.**	**%**	**No.**	**%**	**No.**	**%**
14	60	21.9	46	27.4	106	23.9
23F	42	15.3	15	8.9	57	12.9
6B	33	12.0	20	11.9	53	12.0
3	31	11.3	7	4.2	38	8.6
19F	30	10.9	17	10.1	47	10.6
4	9	3.3	0	0.0	9	2.0
19A	9	3.3	33	19.6	42	9.5
9V	7	2.5	5	3.0	12	2.7
23A	7	2.5	1	0.6	8	1.8
15B	5	1.8	9	5.4	14	3.2
20	5	1.8	2	1.2	7	1.6
Others	37	13.5	13	7.7	50	11.3
Total	275	100	168	100	443	100

There was a significant increase in incidence of serotype 19A (*P* < 0.001), but a significant decline in incidence of serotypes 14, 23F, and 6B over time. For adult patients, the incidence of serotype 19A significantly increased (*P* < 0.001). For children, the incidence of serotype 19A rose from <5% between 2000 and 2008 to 71.4%–75.0% between 2011 and 2012 (*P* < 0.001). The incidence of serotype 3 significantly increased (*P* < 0.001), but that of serotypes 23F and 19F significantly decreased (*P* = 0.027, and *P* = 0.049).

### Serotype distribution in different types of IPD

Blood was the most common source of isolates (n = 370, 83.5%), followed by pleural effusion (n = 46, 10.4%), CSF (21, 4.7%), and ascites (n = 6, 1.4%). Serotype 14 (n = 88, 23.8%) was the most common serotype isolated from blood specimens and serotype 19A (n = 14, 30.4%) was the most common serotype isolated from pleural effusion. In addition, serotype 6B was the most common serotype isolated from CSF and serotype 19F was the most common serotype isolated from ascites specimens.

### Vaccine coverage

**Figure 3:**
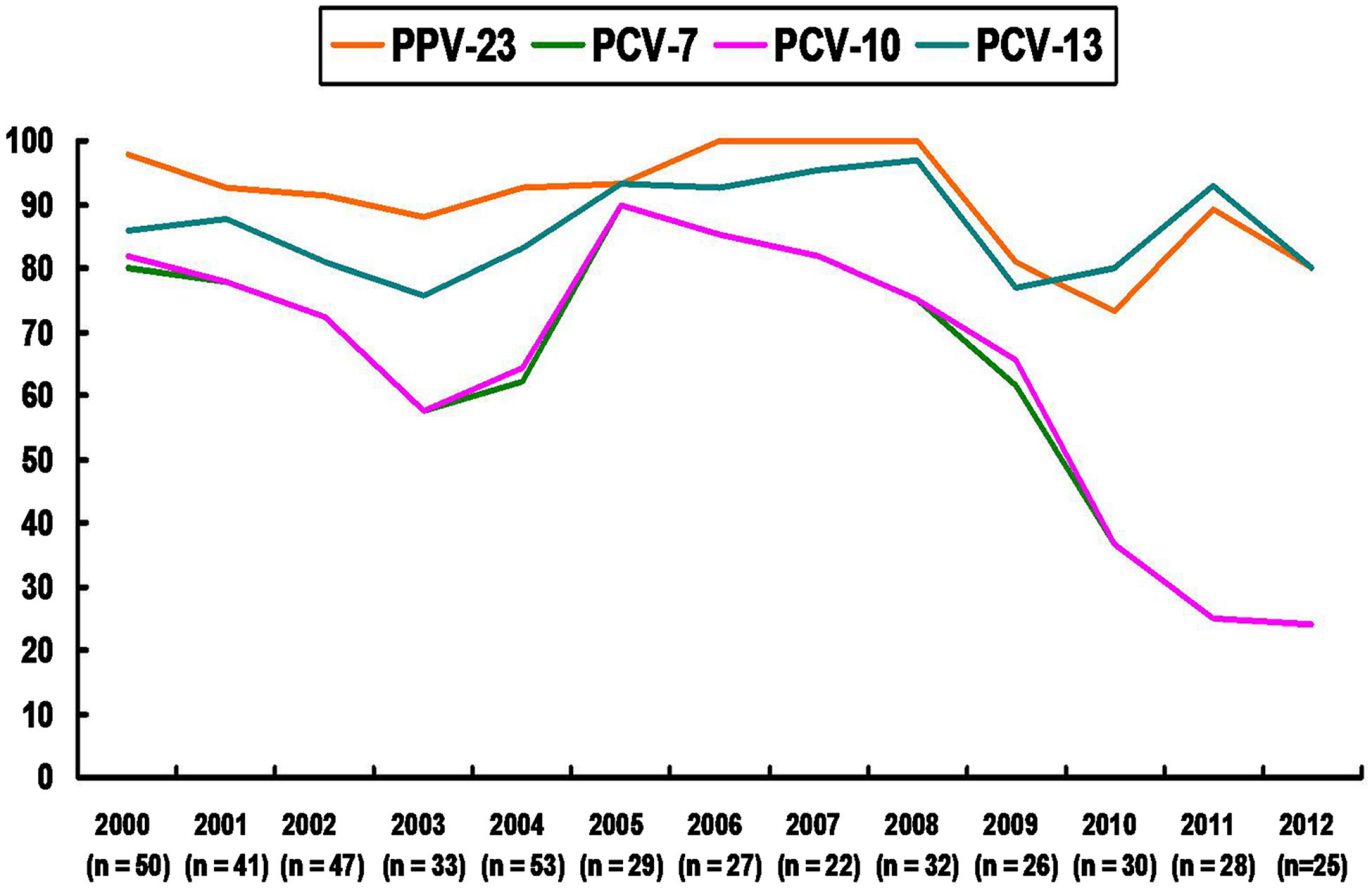
**Coverage rates of different pneumococcal vaccines among *S. pneumoniae *** **isolates recovered from patients with invasive pneumococcal diseases at National Taiwan University Hospital from 2000 to 2012.**

The coverage rates of conjugated vaccines including PCV-7 (PrevnarR; Pfizer), PCV-10 (Synflorix; GSK), PCV-13 (Prevnar 13; Pfizer) and PPV-23 (Pneumovax 23; Merck Sharp & Dohme Corp.) are shown in Figure 3. The overall coverage rates during the period 2000–2012 were 65.2% for PCV-7, 65.9% for PCV-10, 85.8% for PCV-13, and 91.2% for PPV-23. The coverage rates of PCV-7 and PCV-10 significantly decreased with time, but the PCV-13 and PPV-23 coverage rates remained stable. Moreover, PCV-7 and PCV-10 had only a 25% coverage rate in 2012. In contrast, the coverage rates of PCV-13 and PCV-23 were each 80.0% in 2012. The reason coverage of PCV-13 was better than that of PPV-23 in 2010 and 2011 is that there were four serotype 6A isolates found in 2010 and two serotype 6A isolates found in 2011 that were covered by PCV-13 but not by PPV-23.

## Discussion

This study investigated the epidemiology of IPD and the trends and changes in distribution of pneumococcal serotypes in a large Taiwanese hospital over a 13 year period. We found that the incidence of most pneumococcal diseases, including all-cause bacteremia, primary bacteremia, bacteremic pneumonia, peritonitis, and meningitis, significantly decreased with time during study period. In addition, the annual incidence of several pneumococcal serotypes, including serotypes 14, 23F, and 6B significantly decreased with time. In Taiwan, PPV-23 was introduced in 2001 and the cumulative vaccination rate among people aged ≥75 years reached 12% in 2007 and 41% in 2008. PCV-7 was introduced in October 2005 and the cumulative vaccination rate among children aged <5 years was 0.7% in 2005, 8.6% in 2006, 15.9% in 2007, and 25.2% in 2008 [16]. In contrast, PCV-10 and PCV-13 were introduced in Taiwan in 2010, and 2011, respectively. Therefore, our encouraging finding can be attributed to the introduction of PPV-23 and PCV-7. However, we found an increase in the incidence and proportion of serotype 19A among all serotypes causing IPD, especially in children. Although PPV-23 can cover serotype 19A, the use of this vaccine is limited in elderly patients with age above 65 years. Therefore, the emergence of serotype 19A, especially in children in Taiwan may be due to inadequate coverage of vaccination. Moreover, our findings suggest that the secular trend in pneumococcal serotype distribution needs to be closely monitored and that the use of vaccination needs to be adjusted according to the change in serotype distribution.

We found that the coverage rate of PCV-7 and PCV-10 declined from more than 80% in 2000 to 20% in 2012. In contrast, that of PPV-23 and PCV-13 remained stable, ranging from 75% to 97% during the study period. The difference can be attributed to the fact that PPV-23 and PCV-13 cover serotypes 19A and 3. Moreover, PCV-13 can cover serotype 6A but PPV-23 cannot. All of the 8 cases of serotype 6A isolates were found within the last four years (2009 to 2012). Therefore, the clinical impact of PCV-13 for serotype 6A deserves further attention. Based on our findings, PPV-23 and PCV-13 may be a better choice of vaccine to prevent IPD in Taiwan. However, PCV-13 is a newly introduced vaccine in Taiwan; therefore, further surveillance studies are warranted.

We also found that the serotype distribution differed among different diseases. For example, serotype 14 was the most common serotype causing pneumococcal bacteremia in both adults and children [17] and was also the predominant serotype causing empyema in children. In addition to serotype 14, serotypes 3, 6B, 19F, and 23F were the most common serotypes causing bacteremia in adults. In children, serotypes 6B, 19A, 19F, and 23F were common serotypes causing bacteremia. In addition, most cases of meningitis in children and adults were caused by serogroups 6 (n = 7), and 23 (n =5). In contrast, only one patient had serotype 14 meningitis. This finding is consistent with a previously reported finding that serogroups 6, 10, and 23 are more frequently isolated from CSF than serotypes 1, 4, and 14 [17]. Serotype 19F was isolated from 4 of the 6 patients with pneumococcal peritonitis. We also found that some uncommon serotypes were associated with their own distinct syndromes. For example, all patients (n = 9) with serotype 4 and all patients (n = 3) with serotype 22F were adults with bacteremia. In summary, our findings as well as those reported previously [17-20] suggest that some serotypes have a propensity to invade one clinical site rather than another and may therefore be disproportionately responsible for certain disease manifestations. In the present study, serotypes 19A, 14, and 23F were the most common strains causing empyema. This finding differs from that reported in a recent UK study [21], which showed that serotypes 1, 3, 7F, and 19A were most frequently implicated in pneumococcal parapneumonic effusion. This difference may be due to geographical differences.

We also found that the distribution of serotypes differed among different age groups. Although serotype 14 was the predominant serotype in adults and children, the second most common serotype differed between the two age groups. In addition, although serotype 19A was the second most common serotype in children (14.9%), only 2.7% of cases of IPD in adult patients were caused by serotype 19A pneumonococcus. This finding is similar to that recently reported in a study from China, which demonstrated that serotype 19A strains were more frequently isolated from children with IPD [22]. However, our findings differed from those recently reported in a study from Argentina, which showed that serotypes 14, 1, 19A, 5, 12F, 6B, and 18C were the most prevalent serotypes in patients with IPD [23]. In our study, serotype 23A was the second most common serotype in adult patients (15.9%), but only accounted for 9.7% of cases of IPD in children. In addition, serotype 4 only caused pneumococcal infection in nine adult patients. The above-mentioned findings suggest that the distribution of pneumococcal serotypes varies according to different age groups.

This study had one major limitation. We might not have captured all cases of IPD because changes might occur in practices that could have affected rates of culture collection or culture positivity. However, this 13-year longitudinal investigation in a medical center in Taiwan still provided the useful information concerning epidemiology of IPD, and possible impact of pneumococcal vaccination. Most important of all, it might help to guide our government in the implementation of an effective pneumococcal vaccination program.

## Conclusions

Despite we might have missed some cases of IPD in this study, we found that IPD caused by serotype 19A *S. pneumoniae* is an emerging problem in Taiwan, although the incidence of IPD has decreased. The distribution of serotypes of pneumococcal isolates causing IPD varies according to clinical syndromes, and age. As the changing distribution of pneumococcal serotype with time, the coverage rate of each pneumococcal vaccine would be different.

## Competing interests

All authors declare that they have no competing interests.

## Authors’ contributions

CCL draft the manuscript; SHL analyze the data; CHL and WHS collect the data; PRH complete the manuscript. All authors read and approval the final manuscript before submission.

## Pre-publication history

The pre-publication history for this paper can be accessed here:

http://www.biomedcentral.com/1471-2334/14/76/prepub
